# Supplementary material for: The diabetes care continuum in Venezuela: Cross-sectional and longitudinal analyses to evaluate engagement and retention in care
Source: PLOS Glob Public Health. 2024 Jan 17;4(1):e0002763. doi: 10.1371/journal.pgph.0002763 (PMC10793920; doi:10.1371/journal.pgph.0002763)
Supplement: S1 Checklist — (DOCX) [file pgph.0002763.s001.docx]

STROBE Statement—checklist of items that should be included in reports of observational studies

|  | Item No. | Recommendation | Page  No. | Relevant text from manuscript |
| --- | --- | --- | --- | --- |
| **Title and abstract** | 1 | (*a*) Indicate the study’s design with a commonly used term in the title or the abstract | 2 | “This longitudinal study on NCDs is nationally representative at baseline (2014-2017) and has follow-up (2018-2020) data on 35% of participants” |
|  |  | (*b*) Provide in the abstract an informative and balanced summary of what was done and what was found | 2 | See Methods & Results from abstract |
| Introduction | | | |  |
| Background/rationale | 2 | Explain the scientific background and rationale for the investigation being reported | 3-5 | Introduction, but specifically: “Venezuela is a unique case study for NCDs in crises as its socio-political, economic, and nutritional contexts have deteriorated rapidly only in the last decade. ….Shifts in the NCD burden have been challenging to quantify as the Venezuelan government stopped publishing national statistics in 2016.” |
| Objectives | 3 | State specific objectives, including any prespecified hypotheses | 5 | “The specific objectives of this study were to (1) document health system performance for diabetes management in a nationally representative sample of Venezuela in 2014-2017 using the continuum of care framework, (2) assess changes in health system performance over time, from 2014-2017 to 2018-2020, and (3) quantify the association between socio-demographic characteristics and care continuum stage.” |
| Methods | | | |  |
| Study design | 4 | Present key elements of study design early in the paper | 5--6 | “Data are from the EVESCAM study (Estudio Venezolano de Salud Cardio-Metabólica),(23) a longitudinal evaluation of NCDs conducted in Venezuela between 2014 and 2020. EVESCAM has nationally representative data at baseline and follow-up data on a subset of 35% of participants. Details of the study design and sampling strategy have been published elsewhere.(23) Briefly, between July 2014 and January 2017, 4454 study participants were enrolled through a multi-stage stratified sampling method, using parish as the primary sampling unit. Enrolment occurred at the household level, where all members aged ≥20 years were invited to participate; 3420 participants were evaluated for a 76·8% response rate. Exclusion criteria included pregnancy and inability to stand or communicate.(23) The baseline period occurred over three years and enrolment occurred by region, resulting in a strong correlation between time and region. As such, the study was not designed to make any causal claims regarding the effect of the crisis, instead it aimed to provide nationally representative estimates for NCDs.  Between October 2018 and January 2020, study staff contacted and visited every participant enrolled at baseline. If the participant was reachable, study staff collected informed consent and, if provided, continued with clinical measurements and questionnaires as conducted at baseline, along with an updated protocol to measure humanitarian indicators, such as food insecurity, stressful life events, family separation, and access to utilities, medicines, transportation, and education.” |
| Setting | 5 | Describe the setting, locations, and relevant dates, including periods of recruitment, exposure, follow-up, and data collection | 5-6 |  |
| Participants | 6 | (*a*) *Cohort study*—Give the eligibility criteria, and the sources and methods of selection of participants. Describe methods of follow-up  *Case-control study*—Give the eligibility criteria, and the sources and methods of case ascertainment and control selection. Give the rationale for the choice of cases and controls  *Cross-sectional study*—Give the eligibility criteria, and the sources and methods of selection of participants | 5-6 | “Enrolment occurred at the household level, where all members aged ≥20 years were invited to participate; 3420 participants were evaluated for a 76·8% response rate. Exclusion criteria included pregnancy and inability to stand or communicate… Between October 2018 and January 2020, study staff contacted and visited every participant enrolled at baseline. If the participant was reachable, study staff collected informed consent and, if provided, continued with clinical measurements and questionnaires as conducted at baseline, along with an updated protocol to measure humanitarian indicators, such as food insecurity, stressful life events, family separation, and access to utilities, medicines, transportation, and education.” |
|  |  | (*b*) *Cohort study*—For matched studies, give matching criteria and number of exposed and unexposed  *Case-control study*—For matched studies, give matching criteria and the number of controls per case | NA | NA |
| Variables | 7 | Clearly define all outcomes, exposures, predictors, potential confounders, and effect modifiers. Give diagnostic criteria, if applicable | 6-9 | “Blood glucose measurements included fasting plasma glucose (FPG) and a 2-hour oral glucose tolerance test (OGTT) using a 300-ml test solution containing 75 g anhydrous glucose. Diabetes was defined as either: FPG ≥126 mg/dL, 2-hour OGTT ≥200 mg/dL, or self-report of previous diagnosis of diabetes by a clinician.(24) ... SES was calculated using a version of the Graffar Scale modified for Venezuela, which pools income, profession, educational level, and housing conditions into a composite score. Each variable is rated independently from one to five, with one being the highest level of SES. A final score sums the independent ratings and classifies participants’ SES as high, medium-high, medium, medium-low (relative poverty), and low (extreme poverty).(34)” |
| Data sources/ measurement | 8* | For each variable of interest, give sources of data and details of methods of assessment (measurement). Describe comparability of assessment methods if there is more than one group | *6-9* |  |
| Bias | 9 | Describe any efforts to address potential sources of bias | 9 | “Analyses involving the baseline population accounted for complex study design. Analyses that included follow-up measurements were not weighted as the follow-up sample was not representative.” |
| Study size | 10 | Explain how the study size was arrived at | 5-6, 10 | “Details of the study design and sampling strategy have been published elsewhere.(23) … 3420 participants were evaluated for a 76·8% response rate.”, “Weighted multinomial logistic regression models were used to evaluate associations between socio-demographic and clinical characteristics and position on the simplified continuum of care to ensure sufficient sample size (>5) in each stage” |

Continued on next page

| Quantitative variables | 11 | Explain how quantitative variables were handled in the analyses. If applicable, describe which groupings were chosen and why | 9-10 | “All analyses were performed in Stata 17·0 (College Station, Texas, USA). … Predictors included previously well-established risk factors for diabetes,(35) namely, sex (female versus male), age category (<50 years, 50-59 years, ≥60 years), SES (high, medium, and low), urban residence (versus rural), overweight/obesity (versus underweight/normal weight), having hypertension (versus not), or having high LDL (versus not). All models adjusted for participants’ regional residence. The outcome variable was defined as one of three positions on the care continuum (diagnosed, treated, or controlled) compared to undiagnosed.”  “The longitudinal, simplified continuum of care included 210 participants with diabetes at baseline and follow-up data. Individuals with incident diabetes between baseline and follow-up measurements (n=43) were excluded to only analyse individuals already receiving care for diabetes at baseline. Paired t-tests were used to compute statistical differences between the proportions of participants in each stage of the continuum at follow-up compared to baseline. Multinomial logistic regression models were used to examine the association between baseline characteristics and either increasing or decreasing in care continuum stage (vs staying the same). To determine this outcome, participants were given a score at both baseline and follow-up based on their position on the continuum (1 for all diabetes, 2 for diagnosed, 3 for on treatment, and 4 for controlled). The difference between the two scores was calculated and then separated into three categories: worsened, stayed the same, or improved.” |
| --- | --- | --- | --- | --- |
| Statistical methods | 12 | (*a*) Describe all statistical methods, including those used to control for confounding | 9-10 |  |
|  |  | (*b*) Describe any methods used to examine subgroups and interactions | NA |  |
|  |  | (*c*) Explain how missing data were addressed | NA |  |
|  |  | (*d*) *Cohort study*—If applicable, explain how loss to follow-up was addressed  *Case-control study*—If applicable, explain how matching of cases and controls was addressed  *Cross-sectional study*—If applicable, describe analytical methods taking account of sampling strategy | NA |  |
|  |  | (*e*) Describe any sensitivity analyses | NA |  |
| Results | | | | |
| Participants | 13* | (a) Report numbers of individuals at each stage of study—eg numbers potentially eligible, examined for eligibility, confirmed eligible, included in the study, completing follow-up, and analysed | 11, | “Between July 2014 and January 2017, 4,454 participants were recruited and 3,445 participants were available for evaluation. The final sample for analysis included 3,420 adults, and 585 of those individuals had diabetes. Between July 2014 and January 2017, 4,454 participants were recruited and 3,445 participants were available for evaluation. The final sample for analysis included 3,420 adults, and 585 of those individuals had diabetes.’  “Between October 2018 and January 2020, study staff contacted and visited the 3,420 participants enrolled at baseline and collected follow-up data on 1,289 individuals, 210 with diabetes.” |
|  |  | (b) Give reasons for non-participation at each stage | 5 | Due to unique context of this study in a humanitarian crisis, this was not possible to collect for follow-up. At baseline, this information is cited in reference #22 |
|  |  | (c) Consider use of a flow diagram | 5 | Flow diagram cited in reference #22 |
| Descriptive data | 14* | (a) Give characteristics of study participants (eg demographic, clinical, social) and information on exposures and potential confounders | 11, 13 | “Nationally representative socio-demographic characteristics are listed in Supplementary Table 1, pp 2.”  “In the longitudinal sample, most participants with diabetes were female, above 60 years of age, had low SES, and lived in urban areas (Table 2). Most of these participants also had overweight/obesity, hypertension, and high LDL cholesterol” |
|  |  | (b) Indicate number of participants with missing data for each variable of interest | 9 | “Data on sex, age, and SES were missing for <5% of participants.” |
|  |  | (c) *Cohort study*—Summarise follow-up time (eg, average and total amount) | NA | Only two study visits between 2014-2017 and 2018-2020 |
| Outcome data | 15* | *Cohort study*—Report numbers of outcome events or summary measures over time | *13* | “Between 2014-2017 and 2018-2020, 76% of participants gained weight, 9% had no weight change, and 15% lost weight. In 2014-2017, 83% of participants with diabetes who were on treatment were taking oral antidiabetic medications only, 8% were taking insulin only, and 9% were taking a combination. This remained similar in 2018-2020: 81% were taking oral medications only, 7% were taking insulin only, and 12% were taking both.” |
|  |  | *Case-control study—*Report numbers in each exposure category, or summary measures of exposure |  |  |
|  |  | *Cross-sectional study—*Report numbers of outcome events or summary measures |  |  |
| Main results | 16 | (*a*) Give unadjusted estimates and, if applicable, confounder-adjusted estimates and their precision (eg, 95% confidence interval). Make clear which confounders were adjusted for and why they were included | 11, 12 | “Compared to younger participants, older participants were more likely to be on treatment [relative risk ratio (RRR) (95% CI), 2.61 (1·17, 5·81)] and achieve glycaemic control [RRR (95% CI), 2·28 (1·17, 5·81)] compared to being undiagnosed (Table 1). Participants with medium SES were marginally less likely to be on treatment (compared to being undiagnosed) than their counterparts with high SES [RRR (95% CI), 0·39 (0·15, 1·03)]. Finally, women were marginally more likely to achieve glycaemic control than men compared to being undiagnosed [RRR (95% CI) 1·83 (0·95, 3·54)].”  “Multinomial logistic regression was used where the outcome was a 4-level categorical variable: undiagnosed (i.e. in the all diabetes group), diagnosed, on treatment, controlled. Each model controlled for region, one of the above covariates of interest, and weighted for complex survey design.” |
|  |  | (*b*) Report category boundaries when continuous variables were categorized | NA |  |
|  |  | (*c*) If relevant, consider translating estimates of relative risk into absolute risk for a meaningful time period | NA |  |

Continued on next page

| Other analyses | 17 | Report other analyses done—eg analyses of subgroups and interactions, and sensitivity analyses |  |  |
| --- | --- | --- | --- | --- |
| Discussion | | | | |
| Key results | 18 | Summarise key results with reference to study objectives | 17 | “The proportion of people with diagnosed diabetes who were on treatment declined over time, from 60% to 51%. Nonetheless, even in 2018-2020, after over five years of political and economic upheaval, half of people diagnosed with diabetes were treated and nearly two out of five had achieved glycaemic control”. |
| Limitations | 19 | Discuss limitations of the study, taking into account sources of potential bias or imprecision. Discuss both direction and magnitude of any potential bias | 19-20 | “There are several limitations to this study. First, the EVESCAM study experienced high loss-to-follow-up between baseline and follow-up, at 65%. This is expected considering mass emigration and movement within the country. As of December 2022, over 7·0 million Venezuelans had fled their country and there remains no reliable estimates for internal displacement, though the Internal Displacement Monitoring Centre suggest that a displacement crisis is likely based on cross-border movement and conditions inside the country.(20) As shown in Supplemental Table 2, the largest subgroups lost to follow-up in EVESCAM were younger men who had high SES and lived in cities. Therefore, the estimates presented in this paper are representative of those who stayed, a population that is more likely to be female, lower SES, and rural. This aligns with surveys of Venezuelan migrants in Colombia, which recorded a population of primarily men, under 50 years of age, with higher educational attainment but low income, and seeking support for their families remaining in Venezuela.(39) Second, our definition of glycaemic control was based on only one blood glucose measurement at each time point rather than HbA1c , which measures the average glucose levels over the course of red blood cells lifespan (approximately 40-60 days).(40) We calculated HbA1c levels post hoc using the fasting blood glucose measurement, which may have introduced some inaccuracy for prevalence estimates.(40) Finally, two stages of the care continuum – diagnosis and treatment – were based on self-report and could not be confirmed with medical records.” |
| Interpretation | 20 | Give a cautious overall interpretation of results considering objectives, limitations, multiplicity of analyses, results from similar studies, and other relevant evidence | 18-19 | Previous literature on diabetes management in crises underscore the complexity of disease care in these settings. … . Our results suggest that the decline in treatment rates among people diagnosed with diabetes in Venezuela did not differ by SES, urban residence, or age, similarly affecting all population subgroups.” |
| Generalisability | 21 | Discuss the generalisability (external validity) of the study results | 18-20 | “Therefore, the estimates presented in this paper are representative of those who stayed, a population that is more likely to be female, lower SES, and rural. This aligns with surveys of Venezuelan migrants in Colombia, which recorded a population of primarily men, under 50 years of age, with higher educational attainment but low income, and seeking support for their families remaining in Venezuela.” |
| Other information | |  | | |
| Funding | 22 | Give the source of funding and the role of the funders for the present study and, if applicable, for the original study on which the present article is based | 21 | “Funding sources played no role in study design, data collection or analysis, in writing the manuscript or in the paper submission.” |

*Give information separately for cases and controls in case-control studies and, if applicable, for exposed and unexposed groups in cohort and cross-sectional studies.

**Note:** An Explanation and Elaboration article discusses each checklist item and gives methodological background and published examples of transparent reporting. The STROBE checklist is best used in conjunction with this article (freely available on the Web sites of PLoS Medicine at http://www.plosmedicine.org/, Annals of Internal Medicine at http://www.annals.org/, and Epidemiology at http://www.epidem.com/). Information on the STROBE Initiative is available at www.strobe-statement.org.
