# Supplementary material for: The diabetes care continuum in Venezuela: Cross-sectional and longitudinal analyses to evaluate engagement and retention in care
Source: PLOS Glob Public Health. 2024 Jan 17;4(1):e0002763. doi: 10.1371/journal.pgph.0002763 (PMC10793920; doi:10.1371/journal.pgph.0002763)
Supplement: S1 Fig — (DOCX) [file pgph.0002763.s002.docx]

**S1 Fig: Participant Flow Chart**


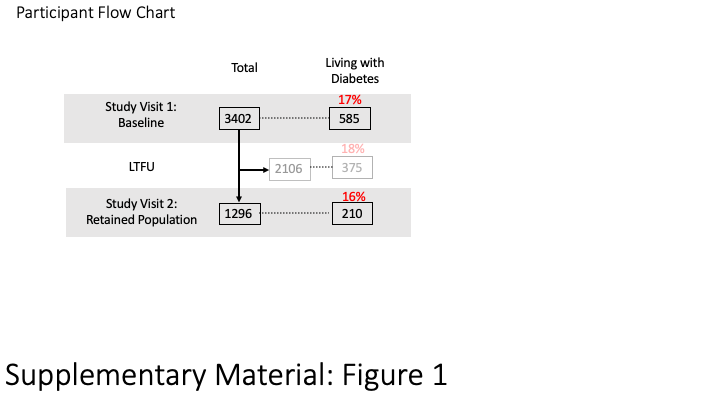


The following flow chart shows (1) the total number of participants in the baseline, lost to follow-up (LTFU), and retained populations, (2) what proportion of each population had diabetes, (3) the number of individuals in each population with diabetes. Diabetes was defined as FPG ≥126 mg/dL, 2-hour OGTT ≥200 mg/dL, or self-report of previous diagnosis of diabetes by a clinician.^28^
