# Supplementary material for: The diabetes care continuum in Venezuela: Cross-sectional and longitudinal analyses to evaluate engagement and retention in care
Source: PLOS Glob Public Health. 2024 Jan 17;4(1):e0002763. doi: 10.1371/journal.pgph.0002763 (PMC10793920; doi:10.1371/journal.pgph.0002763)
Supplement: S1 Table — (DOCX) [file pgph.0002763.s003.docx]

**S1 Table: Baseline sociodemographic and clinical characteristics of 585 Venezuelan adults with diabetes during study period 2014-2017, nationally representative**

|  | **Total (n=585)** | | | **Male (n=212)** | | | **Female (n=373)** | | |  |
| --- | --- | --- | --- | --- | --- | --- | --- | --- | --- | --- |
|  | % | (95% CI) | | % | (95% CI) | | % | (95% CI) | | P-value^1^ |
| **Overall** |  |  |  | 56.6 | (51.7 | , 61.3) | 43.4 | (38.7 | , 48.3) |  |
| **Age** |  |  |  |  |  |  |  |  |  | 0.710 |
| <50 years | 46.3 | (39.6 | , 53.2) | 47.2 | (38.4 | , 56.1) | 47.2 | (38.4 | , 56.1) |  |
| 50-59 years | 22.7 | (18.0 | , 28.1) | 22.5 | (16.2 | , 30.4) | 22.5 | (16.2 | , 30.4) |  |
| 60+ years | 31.0 | (26.2 | , 36.3) | 30.3 | (24.2 | , 37.2) | 30.3 | (24.2 | , 37.2) |  |
| **SES^2^** |  |  |  |  |  |  |  |  |  | 0.395 |
| High | 19.9 | (14.8 | , 26.2) | 22.2 | (15.4 | , 31.0) | 16.9 | (11.9 | , 23.5) |  |
| Medium | 28.0 | (23.5 | , 33.1) | 28.2 | (21.4 | , 36.1) | 27.9 | (22.6 | , 33.9) |  |
| Low | 52.0 | (45.2 | , 58.8) | 49.6 | (39.6 | , 59.7) | 55.2 | (48.4 | , 61.8) |  |
| **Urban** | 81.2 | (60.2 | , 92.5) | 79.6 | (57.1 | , 92.0) | 77.7 | (51.6 | , 92.0) | 0.814 |
| **BMI ≥25** | 73.9 | (65.3 | , 81.0) | 74.6 | (65.4 | , 82.0) | 71.4 | (64.5 | , 77.5) | 0.820 |
| **Hypertension^3^** | 53.9 | (47.5 | , 60.1) | 52.0 | (42.7 | , 61.2) | 58.0 | (50.7 | , 65.0) | 0.507 |
| **High LDL cholesterol^4^** | 59.6 | (54.2 | , 64.8) | 59.4 | (50.3 | , 68.0) | 64.1 | (56.4 | , 71.2) | 0.426 |

These participants had diabetes and were in the baseline sample, i.e. had data for baseline (2014-2017). Unweighted n and weighted percentages are reported.

^1^ P-values calculated using chi-squared tests

^2^ SES was calculated using a version of the Graffar Scale modified for Venezuela, which combines income, profession, educational level, and housing conditions into a composite score**.**

^3^Hypertension was defined as having a systolic blood pressure ≥140 mm Hg, diastolic blood pressure ≥90 mm Hg, or self-report of antihypertensive medication use.

^4^High LDL cholesterol was defined as LDL of ≥100 mg/dL
