# Supplementary material for: The diabetes care continuum in Venezuela: Cross-sectional and longitudinal analyses to evaluate engagement and retention in care
Source: PLOS Glob Public Health. 2024 Jan 17;4(1):e0002763. doi: 10.1371/journal.pgph.0002763 (PMC10793920; doi:10.1371/journal.pgph.0002763)
Supplement: S2 Table — (DOCX) [file pgph.0002763.s004.docx]

**S2 Table: Sociodemographic and clinical characteristics of Venezuelan adults included in total nationally representative population and follow-up with diabetes, at baseline**

|  | **Population excluded from Follow-up, at Baseline** | | **Follow-up Population, at Baseline^1^** | |  |
| --- | --- | --- | --- | --- | --- |
|  | n=365 | % | n=210 | % | P-value^2^ |
| **Female** | 219 | 60% | 145 | 70% | 0.020 |
| **Age** |  |  |  |  | 0.046 |
| <40 | 37 | 10% | 14 | 6% |  |
| 40-49 | 60 | 16% | 22 | 10% |  |
| 50-59 | 88 | 24% | 60 | 27% |  |
| 60+ | 180 | 49% | 123 | 56% |  |
| **SES^2^** |  |  |  |  | 0.011 |
| High | 72 | 20% | 22 | 10% |  |
| Medium | 96 | 27% | 61 | 29% |  |
| Low | 193 | 53% | 129 | 61% |  |
| **Urban** | 323 | 88% | 175 | 80% | 0.005 |
| **Overweight BMI^3^** | 269 | 74% | 152 | 74% | 1.000 |
| **Hypertension^4^** | 238 | 65% | 135 | 65% | 0.942 |
| **High cholesterol^5^** | 219 | 60% | 151 | 73% | 0.219 |

^1^This population only includes individuals who had diabetes during baseline measurements.

^2^ P-values calculated using Chi-squared tests.

^3^ SES was calculated using a version of the Graffar Scale modified for Venezuela, which combines income, profession, educational level, and housing conditions into a composite score**.**

^4^BMI was defined as weight (measured in kilograms) divided by height (measured in meters) squared and classified as overweight/obese (≥25.0 kg/m^2^) or underweight/normal weight (<25.0 kg/m^2^).

^5^Hypertension was defined as having a systolic blood pressure ≥140 mm Hg, diastolic blood pressure ≥90 mm Hg, or self-report of antihypertensive medication use.

^6^High LDL cholesterol (>100 mg/dL)
