# Supplementary material for: The diabetes care continuum in Venezuela: Cross-sectional and longitudinal analyses to evaluate engagement and retention in care
Source: PLOS Glob Public Health. 2024 Jan 17;4(1):e0002763. doi: 10.1371/journal.pgph.0002763 (PMC10793920; doi:10.1371/journal.pgph.0002763)
Supplement: Attachment — Submitted filename: Response to Reviewers_11OCT2023.docx [file pgph.0002763.s005.docx]

| Comment | Response | Page # |
| --- | --- | --- |
| Editorial Comments: | | |
| 1. Please include a complete copy of PLOS’ questionnaire on inclusivity in global research in your revised manuscript. Our policy for research in this area aims to improve transparency in the reporting of research performed outside of researchers’ own country or community. The policy applies to researchers who have travelled to a different country to conduct research, research with Indigenous populations or their lands, and research on cultural artefacts. The questionnaire can also be requested at the journal’s discretion for any other submissions, even if these conditions are not met.  Please find more information on the policy and a link to download a blank copy of the questionnaire here: <https://journals.plos.org/globalpublichealth/s/best-practices-in-research-reporting>. Please upload a completed version of your questionnaire as Supporting Information when you resubmit your manuscript | Done | Uploaded as ‘Other’ document |
| 2. Please send a completed 'Competing Interests' statement, including any COIs declared by your co-authors. If you have no competing interests to declare, please state "The authors have declared that no competing interests exist". Otherwise please declare all competing interests beginning with the statement "I have read the journal's policy and the authors of this manuscript have the following competing interests:" | I have read the journal's policy and the authors of this manuscript have the following competing interests: Dr. Goodman-Palmer provides scientific consultations through Epidemiologic Research & Methods, LLC (ERM); none of her consulting through ERM is related to the topic of the current study. All authors declare that they have no known competing financial interests or personal relationships that could have appeared to influence the work reported in this paper. | 22 |
| 3. Please amend your detailed Financial Disclosure statement. This is published with the article. It must therefore be completed in full sentences and contain the exact wording you wish to be published.  a. State the initials, alongside each funding source, of each author to receive each grant. b. State what role the funders took in the study. If the funders had no role in your study, please state: “The funders had no role in study design, data collection and analysis, decision to publish, or preparation of the manuscript.” c. If any authors received a salary from any of your funders, please state which authors and which funders.  If you did not receive any funding for this study, please simply state: “The authors received no specific funding for this work.” | The follow-up of the Venezuelan Study of Cardiometabolic Health (EVESCAM) was funded by the Harvard T.H. Chan School of Public Health, Departments of Global Health and Population and Epidemiology, Bernard Lown Scholars in Cardiovascular Health Program (BLSCHP-1703). LMJ was supported by the Medical Research Council/UK Research and Innovation, grant number: MR/T044527/1. DGP was supported by NIH T32 HL 098048. For the purpose of open access, the author has applied a Creative Commons Attribution (CC BY) license to any Author Accepted Manuscript version arising from this submission. The funders had no role in study design, data collection and analysis, decision to publish, or preparation of the manuscript. | 22 |
| 4. Please provide separate figure files in .tif or .eps format only and remove any figures embedded in your manuscript file. Please also ensure all files are under our size limit of 10MB | Done | Uploaded all figures as separate files |
| 5. In the online submission form, you indicated that "De-identified data is available upon request". All PLOS journals now require all data underlying the findings described in their manuscript to be freely available to other researchers, either 1. In a public repository, 2. Within the manuscript itself, or 3. Uploaded as supplementary information. This policy applies to all data except where public deposition would breach compliance with the protocol approved by your research ethics board. If your data cannot be made publicly available for ethical or legal reasons (e.g., public availability would compromise patient privacy), please explain your reasons by return email and your exemption request will be escalated to the editor for approval. Your exemption request will be handled independently and will not hold up the peer review process, but will need to be resolved should your manuscript be accepted for publication. One of the Editorial team will then be in touch if there are any issues. | Given the political situation in Venezuela and the vulnerability of some of the participants included in this study, we are not able to make the data publicly available. However, the code used for this manuscript has been posted on the Harvard Dataverse, a public data repository: https://dataverse.harvard.edu/dataset.xhtml?persistentId=doi%3A10.7910%2FDVN%2FRKDD8F&version=1.1 | NA |
| Reviewer #1 | | |
| The authors of the study “The diabetes care continuum in Venezuela: cross-sectional and longitudinal analyses to evaluate engagement and retention in care” attempt to answer an important question – how is the diabetes care continuum impacted by a humanitarian crisis. Using secondary data on self-reported diabetes management questions, anthropometry and biomarkers from a national panel survey, the authors report that 10% achieved ABC control, with the greatest loss in diabetes care occurring at the diagnosis stage. The study is important, given the anticipated rise in humanitarian emergencies globally as a result of climate change and war. The figures present a useful advance over current studies given the potential for longitudinal inference. However, data limitations render many of the findings, although important, of questionable validity and reliability. I share some suggestions below: | Thank you for taking the time to review our manuscript and for recognizing the importance of this work. You point out a significant challenge of collecting data in the context of a humanitarian crisis. We agree there are limitations to interpreting these data, and have outlined those in the Discussion. However, we feel that despite these limitations, the findings are novel and shed light on a previously unstudied vulnerable group of people living with diabetes. | 18-21 |
| 1. The authors present an excellent characterization of the ABC continuum at baseline. However, the non-response at baseline was not discussed (n = 1009, ~ 25%). What are the reasons for this? Were the survey weights adjusted for non-response? | We did not quantify the reasons why the 1009 participants were unable to attend the local evaluation center at baseline. We believe one of the main reasons was a lack of transportation to the health center where the evaluation was performed. We feel it’s worthwhile to point out that for a national population-based study, 76.7% is an excellent response rate. For example, NHANES (the US National Health and Nutrition Examination Survey) has a response rate of just 52% for the interviewed sample and 49% for the examined sample.^[1]^ | NA |
| 2. How was blood glucose measured? Venous/Capillary? Glucometer etc? | Blood glucose was measured in venous blood samples, which is a strength of the study. This detail has been added to the Methods and Discussion. | 6, 20 |
| 3. How was the criterion of 154 mg/dL of fasting glucose decided? The ADA Standards of Medical Care recommend using 80 – 130 mg/dL for preprandial capillary plasma glucose. The estimated average glucose value of 154 mg/dL is typically for comparing random capillary glucose with continuous glucose monitors. | In this study, A1C could not be measured. Initially, we thought of defining glycemic control based on the optimal fasting blood glucose < 100 mg/dl or 130 mg/dl as ADA Standards of Medical Care state. However, we ultimately decided to use the blood glucose that corresponds to an A1C of 7% since it may be a better reflection of glycemic control in this population. To select this point, the correlation between A1C levels and mean glucose levels were based on the international A1C-Derived Average Glucose study (Nathan et al. 2008), which assessed the correlation between A1C and capillary blood glucose measurements in 507 adults (83% non-Hispanic Whites).    (Nathan et al. 2008)  Citations:  American Diabetes Association. Glycemic Targets: Standards of Medical Care in Diabetes. Diabetes Care 2021;44 (Suppl. 1): S73–S84.  Nathan DM, Kuenen J, Borg R, Zheng H, Schoenfeld D, Heine RJ; A1c-Derived Average Glucose Study Group. Translating the A1C assay into estimated average glucose values. Diabetes Care 2008;31:1473–1478. | 7 |
| 4. Why were hypertension and hyperlipidemia included as predictors of diabetes care continuum? The analysis could benefit from a pre-specified hypothesis of what exposure-outcome association the reader may infer. For instance, one disease status being a predictor of disease management should be informed by prior literature. | We are sorry for the confusion. Hypertension and hyperlipidemia were not predictors of diabetes care continuum but rather part of the care continuum itself. Diabetes, as a chronic dysglycemia disease, is associated with hypertension and dyslipidemia, and the ADA Standards of Medical Care include specific goals for blood pressure and lipids since the main morbidity and mortality associated with diabetes is cardiovascular. Including ABC control in the diabetes care continuum is also consistent with a study conducted by Ali et al. that found patients with diabetes were less likely to achieve health targets for several measures of cardiovascular health.  Citation:  Ali MK, Bullard KM, Gregg EW, Del Rio C. A cascade of care for diabetes in the United States: visualizing the gaps. Annals of internal medicine. 2014;161(10):681-9. | NA |
| 5. The outcome variable for the cross-sectional multinomial model is not truly multinomial. Yes – these represent distinct groups of people, but they were sequentially defined. The two options to consider are to better define the categories (Option 1: undiagnosed diabetes, diagnosed-untreated diabetes, diagnosed-treated-uncontrolled diabetes, diagnosed-treated-controlled diabetes) or run 3 separate models (Option 2: undiagnosed vs diagnosed// diagnosed-untreated vs diagnosed-treated// diagnosed-treated-uncontrolled vs diagnosed-treated-controlled). In the current analysis, the meaning of the coefficients is unclear. | We have renamed the categories as suggested throughout the text. | 11&12 |
| 6. The longitudinal analysis of ‘change in status’ is conceptually not as rigorous as the rest of the paper. Could the authors consider using loss-to-follow-up weights and re-run the analysis on the whole sample as a marginal structural model? Alternatively, restrict the analysis to a loss-to-followup adjusted estimation of transition probabilities. | Unfortunately, we do not have data on the reason for loss-to-follow-up and so our ability to do inverse-probability-of-censoring weighting (IPCW) was ruled out. We have conducted a complete case analysis. We apologize as we have consulted with all of our co-authors and are unsure what is meant by ‘restrict the analysis to a loss-to-followup adjusted estimation of transition probabilities.’ We would be happy to consider this upon receiving clarification and possibly a reference to this approach? | NA |
| 7. In the Discussion (e.g. Lines 398), please temper the findings about how there is a potential lack of power to detect subgroup differences. | This is a great point. Thank you. We have revised the sentence such that it now reads:  *Unlike the present study, which found few differences in change of care continuum position by socio-demographic subgroup, though this may have been due to a lack of power with the small sample size, Manne-Goehler et al. found stark differences by subgroup…* | 20 |
| Minor comments:  1. Check line 209 – seems grammatically incorrect 2. Although the word ‘predictors’ is acceptable from a machine learning standpoint, given the nature of the journal and its target audience, I would encourage using ‘covariates’ for any cross-sectional analysis. | 1. Done.  2. Agree. We have revised to ‘covariates’ throughout. | 8, 9 |
| Reviewer #2 |  |  |
| 1. Abstract: What is the use of Confidence intervals signifies here. Better to describe the results in abstract in percentage, and use the 95%CI in the result section. | We have removed the confidence intervals from the abstract. | 2 |
| 2. Keywords: Use the standard keywords using MeSH criteria. | We have updated the keywords to reflect MeSH terms where applicable, i.e. changing ‘health system performance’ to ‘delivery of health care.’ We have retained a couple relevant keywords that are not MeSH terms such as ‘humanitarian crises’ as these have been used in previous studies of this nature. | 2 |
| 3. Line 84: Use easy language. | We have revised the sentence so that it now reads as follows:  *For example, living with diabetes can involve regular access and adherence to medication, monitoring glucose levels, adherence to specific diets, patient education, and regular visits to health facilities.* | 3 |
| 4. Line 86: Numbers between 0-9 should be written in words. Please ensure that across the text. | Done. | Throughout |
| 5. (Health care versus healthcare), please ensure unifying the pattern. | Done. | Throughout |
| 6. Line 104: (upper-middle income) to be (upper middle-income). | Done. | 4 |
| 7. Line 110: (free diabetes drugs,) to be (drugs for diabetes). | Done. | 4 |
| 8. Line 121: (The specific objectives of this study) to be (the primary objective…). | Done. | 4 |
| 9. Line 135: You can omit (elsewhere). | Done. | 5 |
| 10. Line 144: (15th and 29th). | Done. | 6 |
| 11. Lines 162-179: The paragraphs are to be used in discussion rather than methods. You can brief the phrases without wordiness. | We have moved the reference to previous continuum of care studies to the Discussion. | 6, 18-19 |
| 12. Line 201: the reference 32 is not the original citation for BMI. It used BMI only. Please use the original citation for BMI. | We have updated the citation to:  Clinical guidelines on the identiﬁcation, evaluation, and treatment of overweight and obesity in adults—the evidence report. National Institutes of Health, Obes. Res. 6 (Suppl 2) (1998) 51S–209S. | 7 |
| 13. Line 205: Why there is citation here? | We originally included this citation as it validated the instrument in field studies. Per the reviewer’s suggestion, we have removed it. | 8 |
| 14. Lines 208 and 209: The used the citation here for the original Venezuelan study, while you discuss the measurement of LDL, this will create confusion to the readers. | We have removed the citation mentioned by the reviewer and clarified this section as follows:  *Blood tests included total cholesterol, triglycerides, and HDL cholesterol. LDL cholesterol was calculated using Friedewald’s formula.* | 8 |
| 15. Line 217: this is not the original citation for this already known stratification. There is no need for citation here. | Per the reviewer’s suggestion, we have removed it. | 9 |
| 16. Lines 223 and 224 need grammar check. | This has been revised to read:  *Analyses involving the baseline population used survey weights to account for the complex study design.* | 9 |
| 17. (Sociodemographic vs Socio-demographic), and (95% CI vs 95% C.I.) please unify the style. | Done. | Throughout |
| 18. Line 244: (vs) should be written as (versus) or (vs) across the text, and should be unified. | Done. | Throughout |
| 19. Line 254: (pp) could be omitted. | Done. |  |
| 20. Line 255: The use of CI is not understandable here. | The confidence intervals given are for the weighted proportion. | 11 |
| 21. Lines 260-265: Please rephrase in better understandable way. | We have revised this to clarify how both the treatment and achieving glycemic control group were compared to the undiagnosed group. | 11 |
| 22. Across the text: We better use (men and women) instead of (males and females), especially we are dealing here with adults. | Done. | Throughout |
| 23. In any table: please consider every table in the article as an entity by itself, so if you have enough space, you can put the whole words not abbreviated; otherwise if you use abbreviation you have to put an abbreviation legend below the table even if this was a standard abbreviation and was mentioned previously in the text. Please consider this across the tables one by one. | Done. | 1 |
| 24. Line 307: you can omit pp. | Done. | 15 |
| 25. In the methods section: Please mention what define urban versus rural areas in Venezuela. | We have clarified this as follows:  *Urban was defined as population centers with 2,500 or more inhabitants.* | 10 |
| 26. Across the text: Please unify the style of Brackets used. | Edited to make brackets [] for overall brackets and () for the parentheses inside of these brackets. | Throughout |
| 27. Line 365-369: There is no data to support this explanation in the provided text and data. There is no information about how did these adults with diabetes in Venezuela make use of the health facilities. And there was no information about the included cities, and whether they received the financial support to aid the healthcare system there. So, better explanation is needed. | The EVESCAM team conducted a study on which healthcare facilities were accessed by study participants at baseline. We have added this to the Discussion as follows:  *In fact, a previous analysis of EVESCAM participants in 2014-2017 found that when a health service was required, 67.4% attended public health care centres and 21% used private centres (almost 12.5% with insurance coverage and 8.5% out of pocket). (Nieto-Martinez et al. 2021)*  Citation:  Nieto-Martínez R, González-Rivas JP, Ugel E, Duran M, Dávila E, Constantino R, et al. Cardiometabolic risk factors in Venezuela. The EVESCAM study: a national cross-sectional survey in adults. Primary care diabetes. 2021;15(1):106-14. | 17 |
| 28. Please unify the writing style to be all in English not American style according to PLOS G | Done. | Throughout |

1. <https://www.cdc.gov/nchs/hus/sources-definitions/nhanes.htm#:~:text=For%20NHANES%202017%E2%80%932018%2C%20a,49%25%20for%20the%20examined%20sample>.
